# Experimental demonstration of tunable refractometer based on orbital angular momentum of longitudinally structured light

**DOI:** 10.1038/s41377-018-0034-9

**Published:** 2018-07-25

**Authors:** Ahmed H. Dorrah, Michel Zamboni-Rached, Mo Mojahedi

**Affiliations:** 1 Edward S. Rogers Sr. Department of Electrical and Computer Engineering, University of Toronto, Toronto, ON M5S 3G4 Canada; 2 School of Electrical and Computer Engineering, University of Campinas, Campinas – SP, 13083-852 Brazil

## Abstract

The index of refraction plays a decisive role in the design and classification of optical materials and devices; therefore, its proper and accurate determination is essential. In most refractive index (RI) sensing schemes, however, there is a trade-off between providing high-resolution measurements and covering a wide range of RIs. We propose and experimentally demonstrate a novel mechanism for sensing the index of refraction of a medium by utilizing the orbital angular momentum (OAM) of structured light. Using a superposition of co-propagating monochromatic higher-order Bessel beams with equally spaced longitudinal wavenumbers, in a comb-like setting, we generate non-diffracting rotating light structures in which the orientation of the beam’s intensity profile is sensitive to the RI of the medium (here, a fluid). In principle, the sensitivity of this scheme can exceed ~2700°/RI unit (RIU) with a resolution of ~$10^{ - 5}$ RIU. Furthermore, we show how the unbounded degrees of freedom associated with OAM can be deployed to offer a wide dynamic range by generating structured light that evolves into different patterns based on the change in RI. The rotating light structures are generated by a programmable spatial light modulator. This provides dynamic control over the sensitivity, which can be tuned to perform coarse or fine measurements of the RI in real time. This, in turn, allows high sensitivity and resolution to be achieved simultaneously over a very wide dynamic range, which is a typical trade-off in all RI sensing schemes. We thus envision that this method will open new directions in refractometry and remote sensing.

## Laser beam shaping: Dynamic sensors with a petal-powered twist

A technique that generates rotating laser patterns in the shape of flower petals may open up new opportunities in the field of remote sensing. Ahmed Dorrah from the University of Toronto in Canada and colleagues have developed a tunable, high-resolution laser-based device for refractive index sensors. The researchers superimposed Bessel beams to create new light structures with variable petal-like intensity profiles. Because these beams are created by modulating a source’s orbital angular momentum, they possess helical-shaped wavefronts and intensity profiles that rotate along the optical path. The degree of rotation in these petal-like intensity patterns depends on the medium of propagation. Accordingly, the resulting light structures could be reconfigured on-demand to detect refractive index changes between materials such as air, water, and oil with high resolution and wide dynamic sensing range, much larger than conventional optics.

## Introduction

The interactions of light with a medium can be exploited to sense, measure, and study important properties of the medium^1–11^. One important property is the index of refraction, which plays a crucial role in the design and classifications of most optical materials and devices. As such, modalities that can provide accurate, efficient, economical, and dynamic measurements of the index of refraction are always needed. In the past, various approaches to measure the index of refraction have been proposed and utilized. For instance, in laser-based refractive index (RI) sensing, the change in RI in a medium can be inferred from the angle of refraction of the beam in the medium. This has been accomplished in various ways; it was originally done by measuring the critical angle in the medium using a prism^2^ or measuring the displacement of a beam that is obliquely incident on the sample^3^. More recently, other properties of light (e.g., diffraction) have been utilized to measure the index of refraction^4^. Although these previous techniques are relatively simple to implement, they lack reconfigurability, and their resolution is typically limited to ~$10^{ - 3}$ RI unit (RIU).

In some cases, the frequency response can be more informative where a change in RI can be linked to a shift in the transmission spectrum of a broadband source. This has been manifested by relating the RI change to a shift in the transmission spectrum of micro-ring resonators^12,13^, micro-fiber resonators^14–16^, Mach–Zehnder interferometers^17,18^, a shift in surface plasmon resonance^19,20^, or detecting the shift in the reflection spectrum of Fabry–Perot resonators^21,22^. Polarization is another property of light that has been used to diversify the RI measurements, thereby improving the sensing precision^23,24^. Although these later techniques can detect the index of refraction with high resolution (reaching ~10^−6^ RIU), they often suffer from a lack of tunability and reconfigurability and have a narrow dynamic range, high cost, sensitive interfacing and packaging requirements, and complex device fabrication processes.

To overcome these limitations, we propose and demonstrate a novel RI sensing mechanism that leverages on two important but unexplored degrees of freedom of light: light’s orbital angular momentum (OAM) and the ability to pattern light’s intensity along its direction of propagation. We show how these two degrees of freedom can be utilized to generate rotating intensity patterns (in the shape of flower petals), whose orientation and mode profile are sensitive to the medium’s index of refraction. The proposed scheme is laser based: it only requires a laser source to be shaped by a spatial light modulator (SLM) and simply detected by a CCD camera. Hence, it does not require any sophisticated fabrication or packaging. Furthermore, because beam generation is performed using an SLM, which is addressed by a programmable computer-generated hologram (CGH), the proposed scheme is reconfigurable and provides a dynamic sensitivity that can be tuned in real time. With such tunability, a coarse RI measurement can be performed first over a wide range, and the CGH can then be updated to obtain finer measurements with higher resolution. This tunability can address the challenge of simultaneously achieving high sensitivity and a wide dynamic range, which is a typical trade-off in all RI sensing schemes.

### Background

Light beams with OAM possess helical phase-fronts due to their azimuthal phase dependency that follows ${\mathrm{e}}^{{\mathrm{i}}\ell \phi }$, where $\ell$ is the topological charge or winding index of the helical phase-front. In fact, OAM differs fundamentally from the spin angular momentum (SAM) associated with polarization:^25–28^ unlike SAM, which is limited to a value of ±$\hbar$ per photon, OAM can acquire unbounded values of $\ell \hbar$ per photon ($\ell$ is an integer), thus offering additional degrees of freedom. These new degrees of freedom of light, namely, the OAM modes, have been utilized in imaging^26^, optical trapping^29^, material processing^30^, data communications^31,32^, and motion sensing^9,10^. Here, we show, for the first time (to the best of our knowledge), the unexplored advantages of using light’s OAM to measure the index of refraction of a given medium. Such a development can open new directions in refractometry and remote sensing using structured light.

### Concept

OAM beams are characterized by twisted phase-fronts with a singularity in the beam’s center. As such, they carry zero intensity in the beam’s center, whereas their intensity is distributed over a cylindrical surface along the beam’s axis. An OAM beam with topological charge $\ell$ possesses $\ell$ inter-twined helices in its phase-front^25^. For the same value of $\ell$, the amount of phase twist in each helix, over a finite distance, depends on the wavenumber and the RI of the medium. In essence, the helical phase can encounter different amounts of stretching (or compression) if the same beam propagates in different media (with different RI), as shown below. Although characterized by a helical phase-front, when looking at the transverse intensity profiles of an OAM mode, the intensity is distributed over a continuous ring. Hence, the amount of phase helicity is not readily detected by simply looking at the beam’s intensity profile, and its detection typically requires a wavefront sensing apparatus. However, with a judicious superposition of two OAM beams of opposite helicities such that the two OAM beams carry topological charges with opposite signs, it becomes possible to directly map the helicity in the beam’s phase-front to a modulation in the beam’s intensity profile, which can be easily detected by a CCD camera. This occurs as a result of introducing singularities into the phase-front along the azimuthal direction $\phi$, which in turn creates discontinuities in the beam’s transverse intensity profile—often producing intensity patterns in the form of flower petals^33,34^.

When the longitudinal wavenumbers of the superimposed OAM modes are slightly different, the beating between the spatial frequency harmonics will result in a light structure whose intensity profile can rotate along its optical path. The rotation of light’s intensity pattern along its propagation direction has been previously reported in refs^35–39^. The angular orientation of the rotating beam petals is a function of both its propagation distance ($z$) and its *optical* *length*. In other words, at a fixed detection plane along *z*, the beam orientation will also vary if the RI of the medium is changed. This variation in the beam’s angular orientation can be interpreted based on the fact that the angular velocity of the rotating intensity pattern (petals) is directly linked to the amount of its phase helicity, which, in turn, depends on the RI. As such, it is possible to develop a laser-based sensing scheme using OAM modes such that the change in the RI can be linked to the change in the angular orientation of the beam’s transverse intensity profile that, in turn, is easily detected by a CCD camera. In short, at a given transverse plane, by measuring the angular orientation of the rotating intensity pattern (petals) in an unknown medium with respect to its orientation in air (as a reference), the RI of the unknown medium can then be *accurately* measured.

## Results

### Theoretical framework

The rotating intensity pattern ${\mathrm{\Psi }}\left( {\rho ,\phi ,z,t} \right)$ is composed of multiple OAM modes, where each OAM mode $\psi _\ell$ is a superposition of equal frequency co-propagating Bessel beams of different transverse and longitudinal wavenumbers. The resulting waveform, ${\mathrm{\Psi }}\left( {\rho ,\phi ,z,t} \right)$, is thus given by^39^

1 $${\mathrm{\Psi }}\left( {\rho ,\phi ,z,t} \right) = \mathop {\sum }\limits_{\ell = - \infty }^\infty \psi _\ell \\ = {\mathrm{e}}^{ - {\mathrm{i}}\omega t}\mathop {\sum }\limits_{\ell = - \infty }^\infty \mathop {\sum }\limits_{m = - N}^N A_{\ell ,m}J_\ell \left( {k_\rho ^{\ell ,m}\rho } \right){\mathrm{e}}^{{\mathrm{i}}\ell \phi }{\mathrm{e}}^{{\mathrm{i}}k_z^{\ell ,m}z}$$

Each OAM mode $\psi _\ell$ carries a specific topological charge $\ell$ and is composed of $2N + 1$ Bessel beams of equal order (*l*). For the $m{\hbox{-}}{{\rm{th}}}$ Bessel beam in $\psi _\ell$, the transverse wavenumber $k_\rho ^{\ell ,m}$ is related to the longitudinal wavenumber $k_z^{\ell ,m}$ by the consistency relation $k_\rho ^{\ell ,m} = \sqrt {k^2 - \left( {k_z^{\ell ,m}} \right)^2}$. An important property of these OAM modes is that with the superposition of $2N + 1$ Bessel beams with different spatial frequencies, the longitudinal intensity profile of the resultant beam can be modulated along the *z*-direction (i.e., along the beam axis) in a controlled manner^40,41^. This is achieved, in part, by the coefficients $A_{\ell ,m}$ in Eq. (1), which represent different complex weighting factors for each Bessel beam in the superposition, calculated according to

2 $$A_{\ell ,m} = \frac{1}{L}\mathop {\int }\limits_0^L F_\ell \left( z \right){\mathrm{e}}^{ - {\mathrm{i}}\left( {2\pi m/L} \right)z}{\mathrm{d}}z$$

Function $F_\ell \left( z \right)$ in Eq. (2) is the desired longitudinal (axial) intensity profile. With the proper definition of the topological charges $\ell$ and the associated function $F_\ell \left( z \right)$, Eq. (1) can be deployed to generate a rotating OAM light structure with a predefined longitudinal extent that is independent of the transverse beam’s dimensions. The longitudinal control over the beam’s intensity profile is also an important property that will be utilized to extend the dynamic range of the proposed sensor, as discussed in section “Extending the Dynamic Range of Sensing.” Finally, a summation of two (or more) OAM modes with opposite signs for the topological charge $\ell$ transforms the regular rings associated with the Bessel beam’s transverse intensity profile into petal-like shapes whose rotation per RI change can be detected by a CCD camera^39^.

**Fig. 1:**
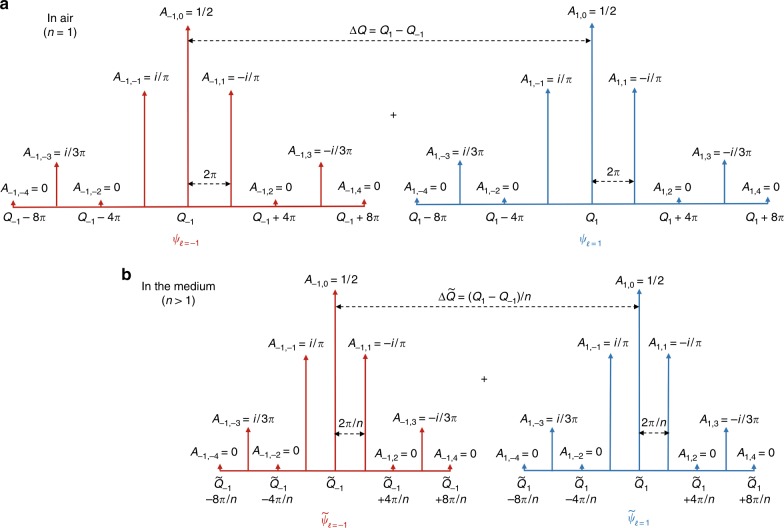
Schematic diagram illustrating the longitudinal wavenumbers of OAM modes $\psi _{ - 1}$and $\psi _1$. **a** Longitudinal wavenumbers in air ($n = 1$). **b** Longitudinal wavenumbers in a medium with unknown refractive index *n*. Here, each OAM mode consists of nine Bessel beams whose longitudinal wavenumbers are equally spaced in a comb-like setting around a constant parameter $Q_\ell$. Additionally, $F_\ell (z) = 1$ for $0{\kern 2pt} {\mathrm{cm}} \le z \le 50{\kern 2pt} {\mathrm{cm}}$ and $\tilde Q \simeq n \times Q$ (see Supplementary Materials for derivation)

The longitudinal wavenumbers of each OAM mode are equally spaced in the *k*-space around a constant parameter $Q_\ell$ in a comb-like setting. More specifically, $k_z^{\ell ,m} = Q_\ell + 2\pi m{\mathrm{/}}L$, where $m \in \left[ { - N,N} \right]$ and *L* is the distance over which the desired profiles are generated. Fig. 1a depicts the spatial frequencies of two OAM modes with opposite helicities, that is, $\psi _{ - 1}$ and $\psi _1$, where the longitudinal wavenumbers are centered at slightly shifted constants, $Q_{ - 1}$ and $Q_1$, respectively. Fig. 1a also depicts the corresponding weighting factors $A_{\ell ,m}$ for each Bessel beam in the superposition of Eq. (1), as obtained with Eq. (2). The phase and amplitude of the coefficients $A_{\ell ,m}$ are evaluated such that the resulting beam extends for 50 cm (as defined by $F\left( z \right)$). This approach allows flexible control over the beam’s range without altering its transverse localization.

For this scenario, $F_\ell \left( z \right)$ was set equal to unity for a distance of 0 cm ≤ $z \le 50$ cm ($\ell = \pm$1) with propagation in air. As the figure shows, the complex coefficients $A_{\ell ,m}$ form a comb-like structure in the *k*-space. Analogous with optical frequency combs, in which the spectral range of frequencies is related to the laser temporal pulse width^43,44^, here, the span of the spatial frequency comb is related to the radial extent of the beam (beam localization). As such, a more radially localized rotating light structure yields a *k*-space comb that spans a wider range of spatial frequencies.

Furthermore, in optical frequency combs, the spectral components are equally spaced in accordance to the laser repetition rate^43,44^, whereas in the case of our *k*-space comb, the teeth are equally spaced by a factor of 2*π*.

A powerful property of frequency combs is their ability to link the precision of optical frequencies with microwave frequencies^44^, thus providing an accurate and precise spectral ruler that can be interfaced (accessed) with electronic circuitry. Here, by interfering two *k*-space combs, we provide a tool that can map the shift in the spatial frequencies of an OAM mode in a given medium, which is also linked to the helicity of the phase-front in that medium, into a rotation in the transverse intensity profile that can be easily detected by a CCD camera. In general, the orientation of the rotating light patterns (petals) resulting from the superposition of two OAM modes, $\psi _{\ell _1}$ and $\psi _{\ell _2}$, is given by^39^

3 $${\mathrm{\Phi }}_{\ell _1,\ell _2} \propto \frac{{\left( {{\mathrm{\Delta }}Q} \right)\left( z \right)}}{{\left| {\ell _1} \right| + \left| {\ell _2} \right|}}$$

where ${\mathrm{\Delta }}Q = Q_{\ell _1}-Q_{\ell _2}$ and *z* is the detection plane. Fig. 1a shows the case when the beam is propagating in air (*n* = 1) and where the longitudinal wavenumbers $k_z^{\ell = 1,m}$ and $k_z^{\ell = - 1,m}$, associated with $\psi _1$ and $\psi _{ - 1}$, are centered around $Q_1$ and $Q_{ - 1}$, respectively, and are equally spaced by a factor of 2*π*. Interestingly, when the same beam is allowed to propagate in a different medium with an unknown RI ($n > 1$), the longitudinal wavenumbers $\tilde k_z^{\ell ,m}$ are shifted and are centered around larger values, $\tilde Q_1$ and $\tilde Q_{ - 1}$, as depicted in Fig. 1b. This is a consequence of the consistency relation $\tilde k_z^{\ell ,m} = \sqrt {k^2 - \left( {k_\rho ^{\ell ,m}} \right)^2}$, in which $k = \omega \frac{n}{c}$, whereas the values of $k_\rho ^{\ell ,m}$ are preserved at the boundary. In a medium with index of refraction *n*, the wavenumbers are still equally spaced but have a smaller spacing, equal to 2$\pi /n$. As Fig. 1b shows, for this case, the spacing between the centers of the two spatial frequency combs becomes ${\mathrm{\Delta }}\tilde Q = \tilde Q_1 - \tilde Q_{ - 1} \simeq {\mathrm{\Delta }}Q{\mathrm{/}}n$ (see Supplementary Materials for derivation).

According to Eq. (3), the change in ${\mathrm{\Delta }}Q$, which is a function of RI, modifies the angular orientation of the beam’s petals (${\mathrm{\Phi }}$). Therefore, in any unknown medium, the rotating intensity pattern exhibits a specific angular orientation ${\mathrm{\Phi }}$ that depends on the RI of the medium. By comparing the angular orientation of the intensity pattern in a medium with an unknown index of refraction (*n*), denoted as ${\mathrm{\Phi }}_{\ell _1,\ell _2}\left( {{\mathrm{\Delta }}\tilde Q} \right)$, with respect to its orientation in air as a reference ($n = 1$), denoted as ${\mathrm{\Phi }}_{\ell _1,\ell _2}\left( {{\mathrm{\Delta }}Q} \right)$, at the same propagation distance, one can then *accurately* determine the RI of the unknown medium.

### Experimental measurements

To examine the performance of the proposed sensing scheme, we generated and tested multiple scenarios in which the rotating structured beams are composed of the superposition of OAM modes $\psi _1$ and $\psi _{ - 1}$, that is, ${\mathrm{\Psi }}\left( {\rho ,\phi ,z = 0,t} \right) = \psi _1 + \psi _{ - 1}$. The beams were generated by an SLM and function $F_\ell \left( z \right)$ was chosen such that

4 $$F_\ell \left( z \right) = \left\{ {\begin{array}{*{20}{c}} {F_1 = F_{ - 1} = 1,\quad0\,{{\rm cm}} \le z \le 50\,{{\rm cm}}} \\ {F_1 = F_{ - 1} = 0,\quad{{\rm elsewhere}}} \end{array}} \right.$$

Using Eq. (3) and the fact that ${\mathrm{\Delta }}\widetilde {\mathrm{Q}} \simeq {\mathrm{\Delta }}Q/n$, it follows that the differential angular orientation of the beam in a given medium with an unknown index of refraction ($n$) with respect to its orientation in air ($n = 1$), denoted as $\theta$ (for $\ell = 1, - 1$), is given by

5 $$\theta = {\mathrm{\Phi }}_{1, - 1}\left( {{\mathrm{\Delta }}Q} \right) - {\mathrm{\Phi }}_{1, - 1}\left( {{\mathrm{\Delta }}\tilde Q} \right) = \frac{{{\mathrm{\Delta }}Q\left( {1 - \frac{1}{n}} \right)z}}{2}$$

**Fig. 2:**
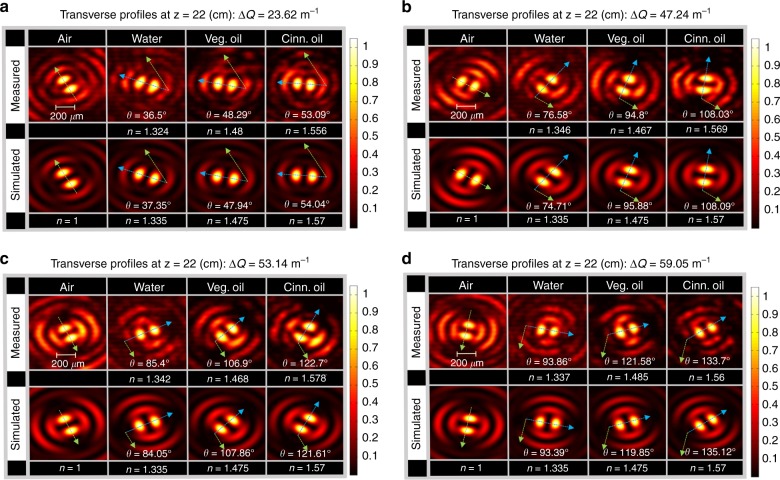
Measured and simulated transverse beam profiles of the rotating beam petals in air, water, vegetable oil, and cinnamon oil at the propagation distance $z = 22\,{\rm cm}$ for different cases of ${\mathrm{\Delta }}Q$. **a** ${\mathrm{\Delta }}Q = 23.62{\kern 2pt} {{\rm m}}^{ - 1}$, **b** ${\mathrm{\Delta }}Q = 47.24{\kern 2pt} {{\rm m}}^{ - 1}$, **c** ${\mathrm{\Delta }}Q = 53.14{\kern 2pt} {\mathrm{m}}^{ - 1}$, and **d** ${\mathrm{\Delta }}Q = 59.02{\kern 2pt} {{\rm m}}^{ - 1}$. The green arrows designate the orientation of the petals in air, and the blue arrows show the orientation of the petals in the medium. The corresponding theoretical and measured quantities ($\theta$ and $n$) are listed for each case

The waveforms were transmitted in air as a reference ($n = 1$), in addition to water ($n = 1.335$), vegetable oil ($n = 1.475$), and cinnamon oil ($n = 1.57$), and then detected at the propagation distance $z = 22$ cm within the medium. The choice of these media was made to offer a wide range of RIs. Fig. 2 illustrates the measured and calculated variations of $\theta$ as a function of the RI of the medium. In each medium, the differential angle $\theta$ represents the orientation of the rotating light petal in that medium with respect to its orientation in air. The measured values of $\theta$ are obtained after identifying the centroids of the detected petal-like structures based on locating the local maxima. From Eq. (5), it can be observed that the sensitivity of this scheme ($\partial \theta {\mathrm{/}}\partial n$) is directly proportional to ${\mathrm{\Delta }}Q$. This is confirmed in Fig. 2a–d, which correspond to cases in which ${\mathrm{\Delta }}Q =23.62, 47.24, 53.14$, and 59.02 m^−1^, respectively. In each case, the unknown index of refraction ($n$) is evaluated with

6 $$n = \frac{1}{{1 - 2\theta /\left( {z{\mathrm{\Delta }}Q} \right)}}$$

**Fig. 3:**
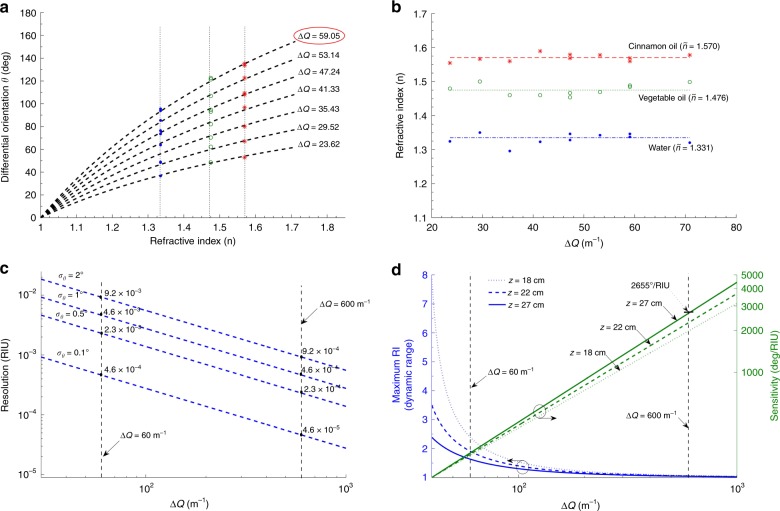
Performance of the proposed sensing scheme at $z = 22$ cm. a Differential orientation (*θ*) as a function of the RI for different values of at $z = 27$ cm, ${\mathrm{\Delta }}Q$ has the units of m^−1^. The vertical dash lines correspond to the refractive indices of water ($1.335$), vegetable oil ($1.475$), and cinnamon oil ($1.57$). **b** Measured refractive indices as a function of ${\mathrm{\Delta }}Q$. The markers represent the measured RI values, and the dashed lines represent the nominal values from the vendor. The averaged measured values for the refractive indices are $1.331$, $1.476$, and $1.570$ for water, vegetable oil, and cinnamon oil, respectively. **c** Resolution of the proposed scheme as a function of ${\mathrm{\Delta }}Q$ under different scenarios of the standard deviation ($\sigma _{\theta}$) in estimating $\theta$. **d** Sensitivity and dynamic range of the proposed scheme as a function of ${\mathrm{\Delta }}Q$ at three different detection planes: $z = 18$, $22$, and $27$ cm

Fig. 3a displays the differential orientation angle $\theta$ as a function of the RI for various values of ${\mathrm{\Delta }}Q$. The markers on the figure correspond to the measured $\theta$ in water (blue), vegetable oil (green), and cinnamon oil (red), where the detection plane is still at $z = 22$ cm. The slope of each curve corresponds to the sensitivity ($\partial \theta {\mathrm{/}}\partial n$). From the figure, it is evident that cases with larger ${\mathrm{\Delta }}Q$ possess larger slopes and thus exhibit higher sensitivity, in agreement with Eq. (5). Furthermore, the measured RIs for each ${\mathrm{\Delta }}Q$ scenario, as evaluated using Eq. (6), are shown in Fig. 3b. Each marker represents an average of at least five independent measurements. The measured RI values averaged over all ${\mathrm{\Delta }}Q$ scenarios in each medium are also listed in the figure, and the nominal values (from the vendor) are shown in the dashed lines. The averaged measured RIs are: $1.331$, $1.476$, and $1.570$ for water, vegetable oil, and cinnamon oil, respectively. The corresponding standard deviations in estimating $\theta$ are $\sigma _\theta = 1.9^ \circ$, $1.474^ \circ$, and $0.644^ \circ$, respectively.

Fig. 3c shows the resolution of the proposed sensing mechanism as a function of ${\mathrm{\Delta }}Q$. The resolution of a sensor is the smallest change in the measurand (here, RI) that can be detected. The resolution ($\sigma _{\mathrm{r}}$) is inversely proportional to the sensor’s sensitivity and directly proportional to the standard deviation of the output variable ($\sigma _\theta$), according to $\sigma _{\mathrm{r}} = \sigma _\theta {\mathrm{/}}\left( {\partial \theta {\mathrm{/}}\partial n} \right)$. For the current setup, the sensor’s resolution is on the order of $10^{ - 3}$ RIU, corresponding to a sensitivity of 270°/RIU. As Fig. 3c indicates, by further improving the optical setup (e.g., a better camera and laser with less noise) and, hence, reducing $\sigma _\theta$ or by increasing the value of ${\mathrm{\Delta }}Q$, the sensor’s resolution can be improved further (reaching $\simeq10^{ - 5}$ RIU). We also note that a larger separation in ${\mathrm{\Delta }}Q$ *typically* implies that longitudinal wavenumbers $k_z^{\ell ,m}$ are also more widely separated in the spatial frequency domain. Through the consistency relation $k_\rho ^{\ell ,m} = \sqrt {k^2 - \left( {k_z^{\ell ,m}} \right)^2}$, a larger separation in $k_z^{\ell ,m}$ implies a wider span in the spatial frequency $k_\rho ^{\ell ,m}$ and, hence, a more localized beam (if $k_\rho ^{\ell ,m}$ acquires large values).

The ability to generate highly localized structured light depends on the SLM’s pixel pitch ${\mathrm{\Delta }}x$, where $k_\rho ^{\ell ,m}$ and ${\mathrm{\Delta }}Q$ are inversely proportional to ${\mathrm{\Delta }}x$. By using commercially available SLMs with a pixel pitch of approximately 4 μm (the SLM used in our experiment has a pixel pitch of 36 μm), it is possible to achieve ${\mathrm{\Delta }}Q \approx 600\,{{\rm m}}^{ - 1}$. Therefore, by using currently available SLM technology, it is possible to achieve resolutions in the order of $10^{ - 5}$ RIU. This can be further improved by an order of magnitude ($\sigma _{\mathrm{r}} \approx 10^{ - 6}$) when using metasurfaces or phase masks to generate beams with higher values of ${\mathrm{\Delta }}Q$.

From Eq. (5), it is clear that in addition to an increasing ${\mathrm{\Delta }}Q$, the sensitivity of our scheme depends on the distance *z* (i.e., increasing $z$ yields higher sensitivity). This is depicted in the green curves of Fig. 3d. In principle, the sensitivity of our scheme can exceed 2600°/RIU at the plane of detection $z = 27$ cm for ${\mathrm{\Delta }}Q = 600\,{{\rm m}}^{ - 1}$ (see section “Improving the sensor’s performance by increasing the interaction length”). In this case, higher sensitivity is achieved at the expense of reducing the sensor’s dynamic range. For the profiles displayed in Fig. 2, the upper bound on the dynamic range is dictated by the value of $n$ associated with $\theta = 180^\circ$, after which the rotating pattern reproduces itself (i.e., becomes degenerate). The dynamic range is evaluated by solving $1{\mathrm{/}}\left[ {1 - 2\pi {\mathrm{/}}\left( {z{\mathrm{\Delta }}Q} \right)} \right]$ and is plotted as blue curves in Fig. 3d as a function of ${\mathrm{\Delta }}Q$ at three different detection planes along $z$. It is observed that in contrast to the sensitivity, the dynamic range decreases at larger values of ${\mathrm{\Delta }}Q$ (and distance *z*). Therefore, there is a trade-off between simultaneously achieving high sensitivity (and high resolution) and maintaining a large dynamic range for sensing. In section “Extending the dynamic range of sensing,” we show how higher OAM modes can be exploited to address this problem.

In all the previous cases, we have presented scenarios in which the rotating beam structures are detected at a fixed plane $z = 22$ cm. In the next section, we show the effect that the detection plane $z$ has on the sensitivity and resolution of the proposed scheme.

### Improving the sensor’s performance by increasing the interaction length

**Fig. 4:**
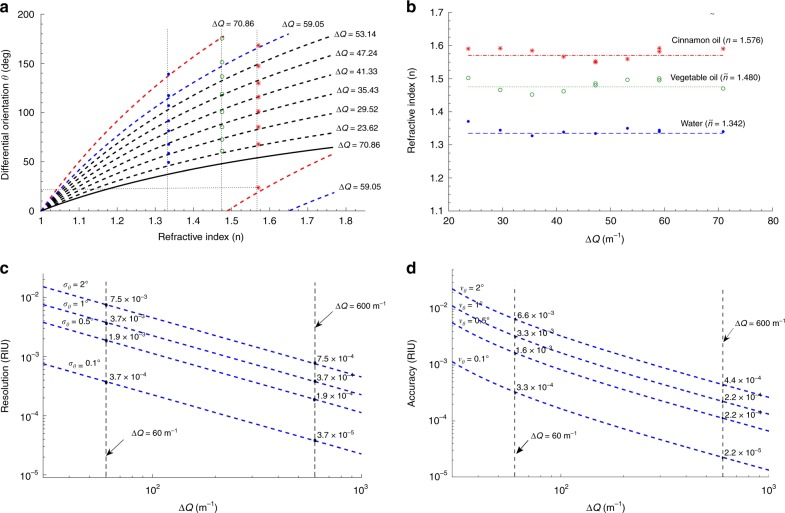
Performance of the proposed sensing scheme at $z = 27$ cm. **a** Differential orientation (*θ*) as a function of the RI for different values of at $z = 27$ cm, ${\mathrm{\Delta }}Q$ has the units of m^−1^.The vertical dash lines correspond to the refractive indices of water ($1.335$), vegetable oil ($1.475$), and cinnamon oil ($1.57$). A degeneracy in detection appears at $n = 1.489$ for ${\mathrm{\Delta }}Q = 70.86{\kern 2pt} {{\rm m}}^{ - 1}$ (red dashed curve). The solid black curve depicts the case of $z = 22$ cm for comparison. **b** Measured refractive indices as a function of ${\mathrm{\Delta }}Q$. The markers represent the measured RIs and the dashed lines represent the nominal values from the vendor. The averaged measured values are $1.342$, $1.480$, and $1.576$ for water, vegetable oil, and cinnamon oil, respectively. **c** Resolution of the proposed scheme at $z = 27$ cm as a function of ${\mathrm{\Delta }}Q$ under different scenarios of the standard deviation *σ*_*θ*_. **d** Accuracy of the proposed sensor at $z = 27$ cm as a function of ${\mathrm{\Delta }}Q$ under different scenarios of the mean absolute error in $\theta$ ($\nu _\theta$)

In addition to the dependence on the spatial frequency separation ${\mathrm{\Delta }}Q$, the sensitivity of the proposed sensing scheme depends on the location of the detection plane along *z*. In the previous cases, we considered a detection plane that was fixed at $z = 22$ cm. Here, we discuss how higher sensitivities can be achieved by setting the detection plane at further distances. Fig. 4a depicts the sensor response ($\theta$) as a function of RI when the detection plane is set at $z = 27$ cm. The markers correspond to the measured $\theta$ in water (blue), vegetable oil (green), and cinnamon oil (red). It is observed that at $z = 27$ cm, the differential angle $\theta$ acquires larger values for the same RI change. Hence, the sensitivity of the scheme can be improved by setting the detection plane at a longer distance along $z$, in agreement with Eq. (5).

The measured RIs at $z = 27$ cm, evaluated from Eq. (6), are plotted in Fig. 4b for each ${\mathrm{\Delta }}Q$ scenario. Each marker represents an average of at least 5 different measurements. The measured RI values, averaged over all ${\mathrm{\Delta }}Q$ scenarios in each medium, are listed in the figure and denoted by $\tilde n$, whereas the nominal values (from the vendor) are shown in the dashed lines. The measured RI at $z = 27$ cm, averaged over all scenarios of ${\mathrm{\Delta }}Q$, are $1.342$, $1.480$, and $1.576$ for water, vegetable oil, and cinnamon oil. The standard deviations in estimating the angular orientation $\theta$ in water, vegetable oil, and cinnamon oil are $\sigma _\theta = 1.24^ \circ$, $1.91^ \circ$, and $1.17^ \circ$, respectively. Again, this uncertainty represents the main limiting factor for the resolution of the proposed scheme.

Fig. 4c depicts the resolution as a function of ${\mathrm{\Delta }}Q$ for different scenarios of *σ*_*θ*_. Resolution is calculated as $\sigma _{\mathrm{r}} = \sigma _\theta {\mathrm{/}}\left( {\partial \theta {\mathrm{/}}\partial n} \right)$, from which the minimum detectable change in the index of refraction ($n$) is obtained. With ${\mathrm{\Delta }}Q = 50\,{{\rm m}}^{ - 1}$ and steps of $\sigma _\theta = 2^ \circ$, the resolution is $7.5 \times 10^{ - 3}$ RIU, which is better than the case of $z = 22$ cm; however, with $\sigma _\theta = 0.1^ \circ$, the resolution can reach $3.7 \times 10^{ - 4}$. Furthermore, the resolution can be improved by an order of magnitude by generating more localized beams in which ${\mathrm{\Delta }}Q$ is ten times larger, as shown in Fig. 4c. For example, the proposed scheme can be utilized to measure RI with a resolution on the order of $10^{-5}$, when ${\mathrm{\Delta }}Q$ is set to $600\,{{\rm m}}^{ - 1}$, which is feasible when using SLMs with a 4 μm pixel pitch.

Accuracy is another important metric in RI sensing. In our proposed scheme, accuracy is determined by the reliable detection of the centroids’ maxima in the rotating petal-like structures. Hence, the error in determining $\theta$ represents the main limiting factor for the accuracy of the proposed scheme. By taking the derivative of Eq. (6) with respect to $\theta$, the accuracy can be expressed as

7 $${\it{\epsilon }}_n = \frac{{2z{\mathrm{\Delta }}Q}}{{\left[ {z{\mathrm{\Delta }}Q - 2\theta } \right]^2}}\nu _\theta$$

where $\nu _\theta$ denotes the mean absolute error in $\theta$. In our experiments, $\nu _\theta$ was ~2° at $z = 27$ cm. Fig. 4d depicts the sensor’s accuracy as a function of ${\mathrm{\Delta }}Q$ under different scenarios of $\nu _\theta$. Similar to the resolution and sensitivity, the sensor’s accuracy can be dramatically improved by using beams with larger values of ${\mathrm{\Delta }}Q$. We also note that because beams with larger values of ${\mathrm{\Delta }}Q$ are more localized, they lead to lower values of $\nu _\theta$. Equation (7) also characterizes the sensor’s precision (i.e., the repeatability of RI measurements over time). This is readily performed by replacing $\nu _\theta$ with the standard deviation ($\sigma _\theta$). A more detailed analysis on the sensor’s tolerance to the deviations in $\theta$, $z$, and ${\mathrm{\Delta }}Q$ can be found in the Supplementary Materials.

### Extending the dynamic range of sensing

**Fig. 5:**
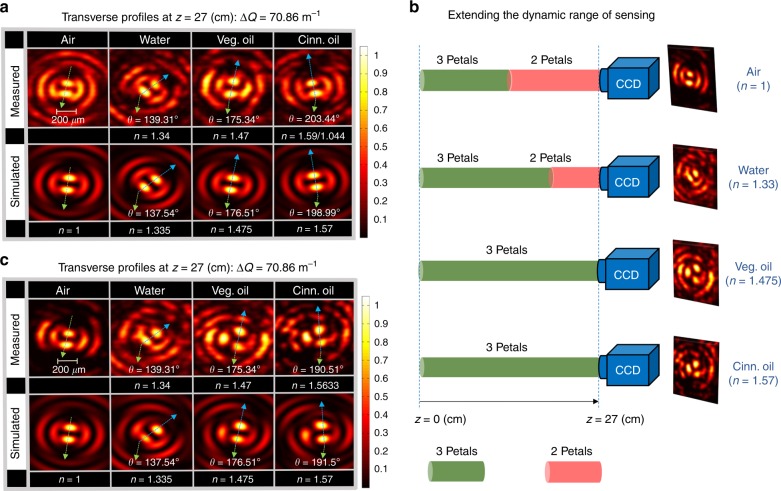
Using higher order OAM modes to extend the dynamic range of sensing. **a** Measured and simulated transverse beam profiles in water, vegetable oil, and cinnamon oil at $z = 27$ cm for ${\mathrm{\Delta }}Q = 70.86{\kern 2pt} {{\rm m}}^{ - 1}$. **b** Schematic diagram showing the evolution of the generated beam from three to two petals with propagation in the different media. **c** Measured and simulated angular orientation of the rotating pattern at propagation distance $z = 27$ cm. The rotating beam is designed to evolve into three petals (instead of two, as in **a**) when the intensity profile is degenerate with the case of air, thus extending the dynamic range of the sensing scheme

In the previous section, we showed that the sensitivity and resolution can be improved by extending the length over which the beam interacts with the medium. This improvement is achieved at the expense of limiting the dynamic range of the sensing scheme. For instance, consider Fig. 5a, which depicts the case when the rotating light pattern, with ${\mathrm{\Delta }}Q = 70.86{\kern 2pt}{{\rm m}}^{ - 1}$, is detected at $z = 27$ cm. It is observed that for vegetable oil, *θ* = $175$°. This implies that the rotating light pattern is very close to acquiring a degenerate orientation compared to a beam that propagates in air. Furthermore, in the case of cinnamon oil, $\theta$ exceeds 180°; hence, mapping the orientation to the index of refraction is no longer unique. In other words, a measured value of $\theta = 203^ \circ$ is degenerate with $\theta = \left( {203^ \circ - 180^ \circ } \right) = 23^ \circ$. Such degeneracy can also be verified from Fig. 4a, as marked by the dashed red curve. It is thus not clear, in this case, if the RI value should be mapped to $1.59$ or $1.044$ (corresponding to the measured orientations $\theta = 203^ \circ$ and $\theta = 23^ \circ$, respectively). As previously mentioned, the dynamic range is constrained when $\theta$ reaches $180^ \circ$, where it can readily be calculated from $1{\mathrm{/}}\left[ {1 - 2\pi {\mathrm{/}}\left( {z\,{\mathrm{\Delta }}Q} \right)} \right]$. This suggests that at ${\mathrm{\Delta }}Q = 70.86\,{{\rm m}}^{ - 1}$ and $z = 27$ cm, the dynamic range of this sensing scheme only spans the range from $n = 1$ to $n \sim 1.489$.

Consequently, there is a clear trade-off between achieving high sensitivity and maintaining a wide dynamic range for RI measurement. However, by incorporating larger topological charges ($\ell > 1$) in the superposition of Eq. (1), it becomes possible to mitigate this trade-off. OAM modes with larger $\ell$ have $\ell$-helices in their phase-front. When these OAM modes—with opposite signs of $\ell$—are superimposed, the rotating pattern will possess more than two rotating petals as a result of introducing additional phase singularities in the azimuthal direction ($\phi$)^39^. In this case, it is possible to generate rotating beams that can change their number of petals (evolving from two to three petals, for instance) as the optical length (index of refraction) is increased. This, in turn, can be utilized to break the degeneracy of the two-petal patterns and extend the dynamic range of the sensor while maintaining high sensitivity and resolution.

For example, consider the waveform ${\mathrm{\Psi }}(\rho ,\phi ,z = 0,t) = \psi _{ - 1} + \psi _1 + \psi _2$, in which the function $F_\ell (z)$ is defined as

8 $$F_\ell \left( z \right) = \left\{ \begin{array}{l}F_{ - 1} = 1,\quad 0{\kern 2pt} {{\rm cm}} \le z \le 50{\kern 2pt} {{\rm cm}}\\ F_2 = 1.5,\quad 0{\kern 2pt} {{\rm cm}} \le z \le 18{\kern 2pt} {{\rm cm}}\\ F_1 = 1,\quad 18{\kern 2pt} {{\rm cm}} < z \le 50{\kern 2pt} {{\rm cm}}\\ F_{ - 1} = F_1 = F_2=0,\quad {{\rm elsewhere}}\end{array} \right.$$

Here, the beam is composed of OAM modes with $\ell = 1$, −1, and 2. As such, the propagating beam (in air) is designed to possess three petals over the range $0{\kern 2pt} {{\rm cm}} \le z \le 18{\kern 2pt} {{\rm cm}}$ before it reduces to two petals over the range $18{\kern 2pt} {{\rm cm}} < z \le 50{\kern 2pt} {{\rm cm}}$. Note that this is the behavior of the beam when propagating  in air. The ability to control the topological charge of structured light along the beam axis is depicted in Fig. 5b, which illustrates the evolution of the beam from three petals to two petals with propagation. This is made possible by the careful constructive and destructive interference among the nine co-propagating Bessel beams in the superposition. With Eq. (8), only OAM modes with non-zero intensity will contribute to the beam center, while the contributions of the other OAM modes are distributed over the outer rings of the beam^39^. Those contributions that are stored in the outer rings can then be restored to the beam’s center at the prescribed locations defined by Eq. (8). In principle, one can discern this effect as a practical manifestation of the Hilbert’s hotel paradox concept^45,46^, in which the spatial redistribution of the local topological charge varies with propagation while the total charge is globally conserved. As the beam is allowed to propagate in different media, the distance over which the beam carries two or three petals changes, depending on the RI (from the relation ${\mathrm{\Delta }}\tilde Q = {\mathrm{\Delta }}Q{\mathrm{/}}n$ and Eq. (3)). Figure 5c represents the measured and simulated transverse intensity profiles of the rotating light structure (with ${\mathrm{\Delta }}Q = 70.86{\kern 2pt} {{\rm m}}^{ - 1}$) in air, water, vegetable oil, and cinnamon oil at a propagation distance of $z = 27$ cm. Here, once the sensing scheme approaches the limit of its dynamic range (i.e., $\theta$ approaching 180°), the same beam evolves into a new intensity profile with three petals instead of two. We note that this evolution occurs without changing the initial hologram on the SLM. In this case, the dynamic range is no longer constrained by the condition $\theta = 180^ \circ$. In other words, there is a one-to-one mapping between the beam orientation and index of refraction of the medium. Accordingly, the dynamic range is now extended from RI = 1 to 2.91, as opposed to the previous case (section “Improving the sensor’s performance by increasing the interaction length”), where the span of dynamic range is from RI = 1 to 1.489. Interestingly, the dynamic range of the beam can be extended even further by incorporating higher OAM modes in the superposition of Eq. (1).

## Discussion

We proposed and experimentally demonstrated a novel tunable RI sensing scheme based on the OAM of light. By adding OAM modes with opposite helicities, we created petal-like light structures that rotate along their optical length. The angular orientation of the rotating petal-like structure depends on the RI of the medium. The rotation in the transverse intensity profile can be measured easily with respect to a reference (air) using a CCD camera, and the differential measurement can then be utilized to accurately identify the RI change. Hence, the proposed sensing scheme is based on a simple setup that only requires an SLM for beam generation and a CCD camera for detection. The sensitivity is only limited by the available SLM bandwidth and can in principle exceed 2700°/RIU with a resolution of $10^{ - 5}$ RIU using SLMs with a 4 μm pixel pitch, which are widely available commercially. We also proposed a novel mechanism to expand the dynamic range of sensing by incorporating higher OAM modes in the transmitted beam, thus scanning the range from RI = 1 to over 2.91. The programmability of the SLM allows the sensitivity, resolution, and dynamic range of the sensor to be reconfigured on demand, thus providing a tunable sensing mechanism that can provide coarse and fine RI measurements in real time.

### Future considerations and outlook

**Fig. 6:**
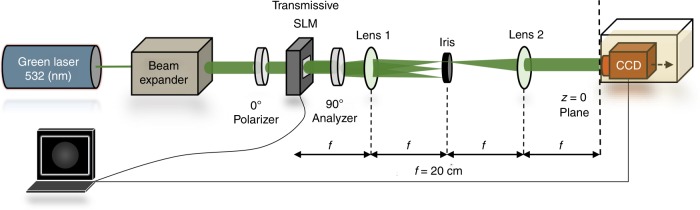
Experimental setup used to generate and detect the structured light used for sensing. A computer-generated hologram (CGH) was sent to a transmissive SLM that encodes the desired pattern on a 532 nm collimated laser beam. The SLM was sandwiched in a polarizer–analyzer configuration as it operates with maximum efficiency on the vertically polarized incident light. The generated pattern was then filtered and imaged using a 4-*f* imaging system. Finally, the beam evolution was recorded inside the fluid using a CCD camera on a translation stage where the $z = 0$ plane lay to the right of Lens 2 (at its focal plane)

In this work, we showed how structured light with OAM can be utilized to measure the real part of the RI. Future considerations include the following: First, the proposed scheme can be deployed to estimate the imaginary part of the RI associated with propagation losses. This can be performed by detecting the intensity level of the measured images. By quantifying the amount of attenuation in the intensity pattern with respect to the reference medium (air) at a given detection distance $z$, the imaginary part of the RI can then be estimated by applying Beer’s law^47^. Since the developed waveform allows control over the longitudinal intensity profile, it is possible to generate rotating light structures that are immune to the propagation losses effects^48,49^. Second, the sensitivity and resolution can be dramatically enhanced by deploying OAM modes with faster rotation rates. This is readily achieved in various ways, such as using accelerated OAM modes^50^ or generating rotating beams with larger values of ${\mathrm{\Delta }}Q$, as explained earlier. Another approach is to replace the second lens in the 4-*f* imaging system of Fig. 6 by a lens with much shorter focal length. This scheme compresses the beam’s longitudinal extent and thus acts as a *photonic gear* that amplifies the beam’s rotation rate along the *z*-direction and, hence, boosts the sensitivity and resolution of the entire scheme. Third, similar to OAM, polarization can be exploited to extend the dynamic range of sensing^51^. Finally, it is also interesting to extend the current sensor to characterize the RI of non-homogeneous media. This will be the subject of future work.

## Materials and methods

To test the proposed sensing mechanism, we performed the following experimental procedure: first, the Bessel beams superposition in Eq. (1) was computed and transformed into 2D transmission CGHs. The holograms were designed for the case of air ($n = 1$) and were sent to a transmissive SLM (Holoeye LC2012). The transmission function at the SLM plane is given by

9 $$H\left( {x,y} \right) = \frac{1}{2} \left\{ {\beta \left( {x,y} \right) + \alpha \left( {x,y} \right){\kern 2pt} {\mathrm{cos}}\left[ {{\mathrm{\Theta }}(x,y) - 2\pi \left( {u_0x + v_0y} \right)} \right]} \right\}$$

Here, $\alpha \left( {x,y} \right)$ and ${\mathrm{\Theta }}(x,y)$ represent the amplitude and phase of ${\mathrm{\Psi }}(\rho ,\phi ,z = 0,t)$, respectively, and $\beta (x,y)$ is a bias function chosen as a soft envelope for the amplitude $\alpha (x,y)$ according to $\beta (x,y) = [1 + \alpha ^2(x,y)]{\mathrm{/}}2$^52^. The pattern is also interfered with a plane wave ${\mathrm{exp}}\left[ {2\pi {\kern 2pt} i\left( {u_0x + v_0y} \right)} \right]$. This superposition shifts the encoded pattern off-axis (in the Fourier plane) to the spatial frequencies ($u_0,v_0$), thus making it easier to filter out the shifted pattern from the undesired on-axis noise with an iris. In our experiment, $u_0$ and $v_0$ were set to $1{\mathrm{/}}(4{\mathrm{\Delta }}x)$, where ${\mathrm{\Delta }}x$ is the SLM pixel pitch (${\mathrm{\Delta }}x = 36$ μm in our case).

Given the twisted nematic nature of our SLM, which makes it operate with maximum efficiency on linearly polarized light, we used a polarizer–analyzer combination oriented at ($0^ \circ$) and ($90^ \circ$) with respect to the SLM axis, as depicted in Fig. 6. The CGH was used to encode the desired pattern on an expanded and collimated green laser beam (532 nm). The generated waveform was then imaged and filtered using a 4-*f* optical system and an iris, which blocked the undesired diffraction orders and on-axis noise. Then, the generated waveform was transmitted into a glass tank at normal incidence. The tank was placed in the focal plane of the 4-*f* imaging system ($z = 0$ plane), as shown in Fig. 6. Finally, the generated waveform was recorded inside the fluid using a CCD camera at a fixed detection plane along *z*. To establish the wide dynamic range capability of our sensing mechanism, we considered the following fluids: (a) deionized water ($n = 1.335$), (b) vegetable oil ($n = 1.475$), and (c) cinnamon oil ($n = 1.57$).

## Electronic supplementary material

Supplemental Material
