# Supplementary material for: Experimental demonstration of tunable refractometer based on orbital angular momentum of longitudinally structured light
Source: Light Sci Appl. 2018 Jul 25;7:40. doi: 10.1038/s41377-018-0034-9 (PMC6107032; doi:10.1038/s41377-018-0034-9)
Supplement: Supplementary file 1 — Supplemental Material [file 41377_2018_34_MOESM1_ESM.pdf]

Supplemental Material for: Experimental demonstration of tunable refractometer based on orbital angular momentum of longitudinally structured light

Ahmed H. Dorrah[1*], Michel Zamboni-Rached[2], and Mo Mojahedi[1]

[1]Edward S. Rogers Sr. Department of Electrical and Computer Engineering,
University of Toronto, Toronto, ON, M5S 3G4, Canada.
[2]School of Electrical and Computer Engineering, University of Campinas,
Campinas, Sao Paulo 13083-852, Brazil.

May 9, 2018

1 Shift of spatial frequencies (k-comb) in the sensed medium

In this section, we derive the relations $\tilde{Q} \simeq n \times Q$ and $\Delta\tilde{Q} = (Q_1 - Q_{-1})/n$, as discussed in section 2.1 of the article. We start from the consistency relation $(k_z^{\ell,m})^2 + (k_\rho^{\ell,m})^2 = k_0^2$ in air and $(\tilde{k}_z^{\ell,m})^2 + (k_\rho^{\ell,m})^2 = k^2$ in the medium, where $k = \omega n/c$. These relations can be re-written as $k_z^{\ell,m} = \sqrt{k_0^2 - (k_\rho^{\ell,m})^2}$ and $\tilde{k}_z^{\ell,m} = \sqrt{k^2 - (k_\rho^{\ell,m})^2}$. Note also that $k_z^{\ell,m} = Q_\ell + 2\pi m/L$ in air. Without loss of generality, let us consider the central term $k_z^{\ell,m=0}$ such that

$$Q_\ell = k_z^{\ell,m=0} = k_0\sqrt{1 - \left(\frac{k_\rho^{\ell,m=0}}{k_0}\right)^2} \tag{S1}$$

In the paraxial regime, $k_0 \ll k_\rho^{\ell,m=0}$, the above expression can be expressed as

$$Q_\ell = k_z^{\ell,m=0} = k_0\left[1 - \frac{1}{2}\left(\frac{k_\rho^{\ell,m=0}}{k_0}\right)^2 - \frac{1}{8}\left(\frac{k_\rho^{\ell,m=0}}{k_0}\right)^4 + ...\right] \tag{S2}$$

Similarly,

$$\tilde{Q}_\ell = \tilde{k}_z^{\ell,m=0} = k_0 n \left[1 - \frac{1}{2}\left(\frac{k_\rho^{\ell,m=0}}{k_0 n}\right)^2 - \frac{1}{8}\left(\frac{k_\rho^{\ell,m=0}}{k_0 n}\right)^4 + ...\right] \tag{S3}$$

The spacing between the central longitudinal wavenumbers in air and in the medium, denoted as ΔQ and $\Delta\tilde{Q}$, are thus given by

$$\Delta Q_\ell = Q_\ell - Q_{-\ell} = k_0 \left[\frac{1}{2}\left(\frac{k_\rho^{-\ell,m=0}}{k_0}\right)^2 - \frac{1}{2}\left(\frac{k_\rho^{\ell,m=0}}{k_0}\right)^2 + \frac{1}{8}\left(\frac{k_\rho^{-\ell,m=0}}{k_0}\right)^4 - \frac{1}{8}\left(\frac{k_\rho^{\ell,m=0}}{k_0}\right)^4 + ...\right] \tag{S4}$$

and

$$\Delta\tilde{Q}_\ell = \tilde{Q}_\ell - \tilde{Q}_{-\ell} = k_0 n \left[\frac{1}{2}\left(\frac{k_\rho^{-\ell,m=0}}{k_0 n}\right)^2 - \frac{1}{2}\left(\frac{k_\rho^{\ell,m=0}}{k_0 n}\right)^2 + \frac{1}{8}\left(\frac{k_\rho^{-\ell,m=0}}{k_0 n}\right)^4 - \frac{1}{8}\left(\frac{k_\rho^{\ell,m=0}}{k_0 n}\right)^4 + ...\right] \tag{S5}$$

By only neglecting the higher order terms (raised to the fourth power) in Eq. (S4) and Eq. (S5), the relation $\Delta\tilde{Q} \simeq (Q_1 - Q_{-1})/n$ is established. Furthermore, by neglecting all the higher order terms (raised to the power of two and four) in Eq. (S2) and Eq. (S3), it follows that $Q \simeq k_0$ and $Q \simeq k_0 n$; thus the relation $\tilde{Q} \simeq n \times Q$ holds true.

2 Sensor's tolerance to deviations in θ, ΔQ, and z

In this section, we characterize the sensor's tolerance to the deviations in the angular orientation (θ), in the separation ΔQ, and in the detection plane (z), using a closed form expression. We start from Eq. (6) in the main article, which states that

$$n = \frac{1}{1 - 2\theta/(z\Delta Q)}. \tag{S6}$$

By taking the partial derivatives with respect to θ, z, and ΔQ, one can then establish the following relation

$$\delta n = \frac{2z\Delta Q}{[z\Delta Q - 2\theta]^2}\delta\theta + \frac{2\Delta Q\theta}{[z\Delta Q - 2\theta]^2}\delta z + \frac{2z\theta}{[z\Delta Q - 2\theta]^2}\delta\Delta Q. \tag{S7}$$

Equation (S7) shows that the proposed sensing scheme is more tolerant to the deviations in the angular orientation, denoted by ($\delta\theta$), when either the parameter ΔQ or the detection plane (z) are set to larger values. Stated otherwise, by generating OAM modes with larger separation ΔQ and increasing the interaction length z, our sensing scheme records smaller errors when identifying the refractive index (n), for the same deviation in θ. This is readily discerned from the quadratic dependency on ΔQ and z in the denominator of Eq. (S7). Hence, at larger ΔQ and/or z, the accuracy and precision of the proposed sensor is improved and it becomes more tolerant to errors in estimating θ as well as the uncertainty in z.
